# Hyperthyroidism with dome-and-dart T wave: A case report: A care-compliant article

**DOI:** 10.1097/MD.0000000000006060

**Published:** 2017-02-10

**Authors:** Ping Lai, Jing-ling Yuan, Jin-hua Xue, Yue-qun Qiu

**Affiliations:** a Gannan Medical University, Ganzhou; b Department of Pathophysiology, School of Basic Medical Sciences, Southern Medical University, Guangzhou; c Department of Physiology, School of Basic Medical Sciences, Gannan Medical University; d Department of Cardiology, Ganzhou Cardiology Center, Society for Cardiovascular Internal Medicine, Emergency Medicine, the First Affiliated Hospital of Gannan Medical University, Ganzhou, China.

**Keywords:** arrhythmia, dome-and-dart T wave, hyperthyroidism

## Abstract

**Rationale:**

Dome-and-dart T waves (or bifid T waves) are a rare phenomenon in the surface electrocardiogram. These wave forms are mainly observed in patients with congenital heart disease such as atrial septal defect and ventricular septal defect. And hyperthyroidism who presented with an electrocardiogram that had dome-and-dart T waves in a precordial lead is never been reported.

**Patient concerns:**

The patient presented with continuous tachycardia, palpitations, chest tightness, and headache for 4 days, and aggravated for 1 day.

**Diagnoses:**

Hyperthyroidism.

**Interventions:**

Methimazole.

**Outcomes:**

All symptoms were alleviated.

**Lessons:**

Dome-and-dart or bifid T waves have been reported in the conventional 12-lead electrocardiograms in some patients with congenital heart disease. The case illustrated here, to the best of our knowledge, dome-and-dart or bifid T waves may associate with hyperthyroidism patients.

## 1 Introduction

Hyperthyroidism is a rare disease in adolescents that can have significant effects on the heart. Hyperthyroidism is easily misdiagnosed as myocarditis because patients can present with tachycardia, hypertension, palpitations, and chest tightness,^[1]^ but without specific clinical manifestations on the surface electrocardiogram. This case report describes our findings in an adolescent patient with hyperthyroidism who presented with an electrocardiogram that had dome-and-dart T waves in a precordial lead.

## 2 Case report

**Figure 1:**
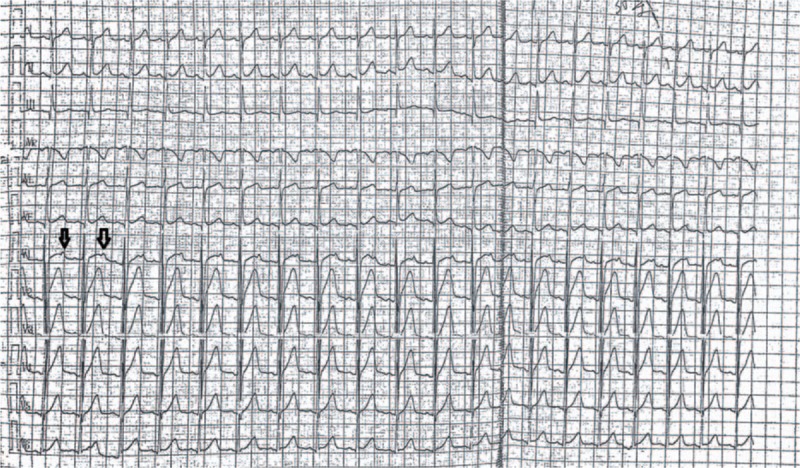
Patient's electrocardiogram before treatment (note the dome-and-dart T wave in V1).

A 15-year-old boy was referred to our hospital for evaluation of continuous tachycardia, palpitations, chest tightness, and headache that had been present for 4 days, and aggravated for 1 day. His heart rate was 105 beats per minute and there were no positive findings from the relevant physical examination. His medical history, family history, and psychosocial history were noncontributory and included no comorbidities, or relevant genetic information. His electrocardiogram showed a P-R interval of 120 milliseconds, a Q-T interval of 330 milliseconds. In lead V1, there were ST-T abnormalities with an initial dome and followed by dart T waves the amplitude of the dart T wave was ≥0.1 mV (Fig. 1).

Echocardiography showed that the atrioventricular cavities were within normal limit and that the structure of the heart chambers structure had no obvious anomalies (left atrial diameter 25 mm, left ventricular diameter 41 mm, right atrial diameter 30 mm, right ventricular diameter 20 mm). Blood tests on admission showed low levels of thyroid-stimulating hormone (TSH, 0.02 μU/mL) and high levels of free thyroxine (T4 > 100.00 pmol/L) and free triiodothyronine (T3 > 50.00 pmol/L). The results of clinical biochemistry blood tests, including myocardial enzymes, troponin, myoglobin, electrolyte serum levels (K^+^ 4.23 mmol/L, Na^+^ 140.6 mmol/L, Ca^2+^ 2.77 mmol/L, Mg^2+^ 0.92 mmol/L) and high sensitivity C-reactive protein (hs-CRP) were within normal limits.

**Figure 2:**
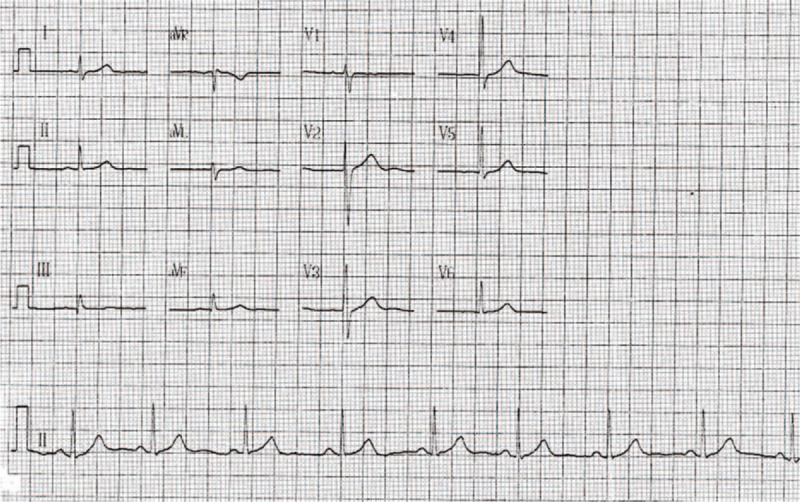
Patient's electrocardiogram after treatment with methimazole for a month (note that the dome-and-dart T wave disappeared in V1).

The patient was diagnosed with hyperthyroidism and treated with methimazole. When seen during follow-up after 1 month of therapy, he was asymptomatic, with no palpitations or headache and had no positive findings on physical examination. Blood tests were rechecked and his clinical biochemistry and blood analysis results were within normal limits, with a TSH of 2.12 μIU/mL, free triiodothyronine >5.32 pmol/L, and free thyroxine >14.3 pmol/L. The 12-lead electrocardiogram at the follow-up visit showed that the dome-and-dart T wave had disappeared (Fig. 2).

## 3 Discussion

Dome-and-dart or bifid T waves have been reported in the conventional 12-lead electrocardiograms in some patients with congenital heart disease. These waveforms are characterized by an initial “dome” portion followed by a “dart” in the descending T wave recorded in right chest leads V1–V3 or V3R.^[2]^ This rare finding in the electrocardiogram is associated with congenital heart disease and has never been reported in a hyperthyroidism patients.

The T wave represents the electric potential variations that occur during ventricular repolarization. Ventricular repolarization can be affected by right ventricular hypertrophy, caused by the increased right ventricular load caused by left to right shunts in patients with congenital heart disease. The first peak of the T wave (the “dome”) reflects the ventricular repolarization wave from the inferior–posterior left ventricular myocardium such that the vector of the T wave is to the right and the forward, projected to the positive side in the right thorax, but the amplitude of the T wave vector is small. The vector of the T wave is forward and to the right and the amplitude is increased in right ventricular hypertrophy, as a second peak follows the first peak of the T wave. But the timing of the “dart” component of the T wave is not delayed as the repolarization time of the right ventricular is not extended.^[3]^

The function of various ion channels in the myocardium can be altered in patients with hyperthyroidism. Studies have shown that, thyroid hormone can affect the regulation of myocardial intracellular calcium in cardiac cells, and promote cytoplasmic calcium resorption by the sarcoplasmic reticulum calcium pump.^[4]^ Some studies have suggested that thyroid hormones have effects on ventricular dynamic repolarization via altered sympathetic activity, and that these effects disappear with normalization of the thyroid hormone levels.^[5]^ Some earlier studies found that high levels of thyroid hormone weakened the long-lasting Ca^2+^ current. Hyperthyroidism promotes the gene expression of voltage-gated potassium ion channels and increases the transient outward potassium current (Ito), and delayed the rectifier current (IKur). In guinea pig cardiac myocytes, an acute increase of triiodothyronine increases the probability of IK_l_ current channel opening. This change of the ion channel function can affect the repolarization phase of myocardial cells.^[6,7]^ Thyroid hormones also cause various arrhythmias by shortening the cell's refractory period of myocardial cells by altering the activity of Na^+^–K^+^ exchange across the cell membrane.^[8]^ Therefore, thyroid hormone levels may have an effect on the Q-T (340–440 milliseconds) and QTc (=Q-T/R-R, 320–440 milliseconds) intervals. However, the mechanisms by which thyroid hormones affect the heart can be complex.

Hyperthyroidism is a rare disease in adolescents. It is easy to misdiagnose as myocarditis, because the clinical signs and symptoms of hyperthyroidism include tachycardia, hypertension, palpitations, and chest tightness, and because it lacks other specific clinical manifestations.^[1]^ This patient was hard to diagnose, as his syndrome was mainly characterized by headache and shortness of breath, and there were no typical clinical manifestations of hyperthyroidism. The electrocardiogram just showed sinus tachycardia, with a dome-and-dart T wave in lead V1, while the dome-and-dart T wave on lead V1 disappeared after treatment of the hyperthyroidism for 1 month. Dome-and-dart T waves have not been reported to be previously associated with hyperthyroidism, which frequently, however, involves atrial fibrillation, premature beats, or cardiac dilatation after long-term exposure to high thyroid hormone levels. The clinical treatment of hyperthyroidism can be difficult and the prognosis poor. Early detection of the effects of excess thyroid hormone on the heart and timely treatment are essential. As hyperthyroidism that presents with cardiovascular complications can be fatal, it is necessary to screen for thyroid dysfunction when patients present with appropriate symptoms, and it now may be necessary to add dome-and-dart T wave in electrocardiogram to those indications. However, we must recognize that the relationship of dome-and-dart T waves to hyperthyroidism remains to be evaluated by further research.

## Acknowledgments

We are grateful for the efforts of Dongming Xie, MS, Department of Cardiology, the First Affiliated Hospital of Gannan Medical University, Ganzhou, China and Yuan-hui Liu, Department of Cardiology, Guangdong Cardiovascular Institute, Guangdong Provincial Key Laboratory of Coronary Heart Disease Prevention, Guangdong General Hospital, Guangdong Academic of Medical Sciences, Guangzhou, China.
